# Optical coherence tomography angiography using the black-and-white pixel binarization histogram software: a new technique for evaluating healing of macular holes in two surgical techniques

**DOI:** 10.1186/s40942-020-00229-w

**Published:** 2020-07-01

**Authors:** Oswaldo Ferreira Moura Brasil, Mariana Kawamuro, Denise Pardini Marinho, Bruna Trench Maia, Murilo Ubukata Polizelli, Gabriel Pipolo, Natalia Trench Maia, Rodrigo Dompieri, Natasha Ferreira Santos da Cruz, Mauricio Maia

**Affiliations:** 1 Department of Ophthalmology, Federal University of São Paulo, São Paulo, Brazil; 2 Brazilian Institute of Ophthalmology (IBOL), Rio de Janeiro, RJ Brazil; 3 Brazilian Institute of Fight Against Blindness (INBRACE), Assis and Presidente Prudente, São Paulo, Brazil; 4 Vision Institute (IPEPO), São Paulo, Brazil

**Keywords:** Optical coherence tomography angiography, Macular hole surgery, Pixel binarization

## Abstract

**Background:**

Many factors can influence the functional outcomes of macular hole surgery and some studies have tried to describe anatomical features that could predict successful treatment. The purpose of this study is to describe a new technique for evaluating the healing of macular holes after two surgical techniques using a black-and-white pixel binarization histogram software by optical coherence tomography angiography and its potential functional implications.

**Methods:**

This was a retrospective, observational case series of patients who presented with idiopathic full-thickness macular holes and underwent vitreoretinal surgery for successful macular hole closure using the internal limiting membrane peeling technique or the inverted peeling technique or the free internal limiting membrane flap technique. Optical coherence tomography angiography 3.0 × 3.0-mm scans were obtained postoperatively. The outer retina layer was analyzed separately; three different analyses were performed within a 3.0-mm-diameter area central circle, a 1.5-mm-diameter area, and a 0.5-mm-diameter area from the foveal center. The outer retinal layer images were evaluated by counting the number of black and white pixels. The inclusion criteria were patients with idiopathic macular holes who underwent ophthalmologic examinations and swept-source optical coherence tomography 1 week and 1 and 6 months postoperatively. The exclusion criteria were a traumatic macular hole or a history of eye trauma and a diagnosis of glaucoma or any other chronic ocular disease. The Mann–Whitney test was used to determine significance; P < 0.05 was considered significant.

**Results:**

Ten eyes of 10 patients who underwent vitreoretinal surgery to treat a macular hole either with the conventional peeling technique (n = 5) or the inverted/free internal limiting membrane flap technique (n = 5) were included. In the peeling group, the percentage of white pixels ranged from 7.22% to 18.40% in the 0.5 × 0.5-millimeter area in the macular center; the mean postoperative logarithm of the minimum angle of resolution best-corrected visual acuity was 0.3 ± 0.2. In the inverted flap group, the percentage of white pixels ranged from 3.65% to 8.93% in the 0.5 × 0.5-millimeter area in the macular center; the mean logarithm of the minimum angle of resolution best-corrected visual acuity was 0.9 ± 0.4.

**Conclusions:**

A simple method of optical coherence tomography angiography imaging analysis of the outer retina using a binarization technique of the black and white pixels was created and may have functional implications during the analysis of the healing process after macular hole surgery. We hypothesized that higher numbers of white pixels correspond to viable cellularity and better prognoses postoperatively.

## Background

Macular holes (MHs) are total thickness defects of the neurosensory retina located in the fovea [1, 2]. The prevalence of this disease varies widely in the literature, ranging from 0.2 cases/1000 inhabitants in the Blue Mountains Study to 3.3 cases/1000 inhabitants in the Baltimore Eye Study. A US population study conducted in Minnesota reported an incidence of 7.8 cases/100,000 inhabitants [2]. The disease occurs more frequently in women in a ratio of 6:1 compared to men, especially after the sixth decade of life, and is bilateral in 10% to 30% of eyes [3, 4].

Although the pathogenesis of MH is not fully understood, it is believed that vitreoretinal traction due to incomplete detachment of the posterior vitreous causes the formation of foveal cysts that, with the maintenance of traction, leads to development of MHs. In addition, tangential vitreoretinal tractional forces and changes in the retinal layers also are implicated in the pathogenesis of this disease [4]. According to the Gass theory, MHs would not result from loss of photoreceptors but the centrifugal displacement of these cells. This could explain the visual recovery that occurs in many of these cases after MH surgery. However, more recent histologic studies and optical coherence tomography (OCT) findings have shown that in some cases significant amounts of foveal tissue, including cones, are lost [5].

Patients with MHs complain of low best-corrected visual acuity (BCVA), central scotomas, and typical metamorphopsia caused by the centrifugal displacement of the photoreceptors. Ophthalmoscopy and OCT confirm the diagnosis and vary according to the stage of the lesion [3, 6].

In 1991, Kelly and Wendel performed pars plana vitrectomy (PPV) combined with an air-fluid exchange in 52 patients with idiopathic MHs and achieved a success rate of about 60%. Among the 30 patients with MH closure, the BCVA improved in 22 patients and worsened in only one. Since then, innovations in surgical techniques have achieved success rates of almost 100% in recent (1–4 weeks) stage 2 and 3 MHs and more than 90% in large stage 4 holes [1, 4]. In 1994, the Trial Vitrectomy for Prevention of Macular Hole excluded the possibility of any benefit from vitrectomy in impending MHs; therefore, surgery is currently indicated for full-thickness MHs (stages 2, 3, and 4 in the Gass classification) [5]. The surgical techniques for treating MHs have improved, and the removal of the internal limiting membrane (ILM) is an important advance resulting in anatomic success exceeding 90% [4].

The state-of-the-art surgery for MH repair typically consists of PPV, separation, and removal of the posterior vitreous cortex, removal of the ILM, filling of the vitreous cavity with gas or air, and postoperative face-down positioning [4]. To aid the ILM peeling, numerous dyes are currently available such as indocyanine green, infracyanine green, lutein-based dyes and brilliant blue (BB); the last is used most frequently worldwide for ILM staining due to its high affinity for this retinal structure and its excellent safety profile when used at the concentration of 0.25 mg/ml [7].

Variations in the standard vitrectomy technique have been described recently. In 2010, Michalewska and colleagues [8] conducted a prospective clinical trial to compare traditional surgery with an ILM flap inversion technique. In the latter, the peeling was performed without complete detachment of the ILM from the retina and the ILM was then inverted over the MH. MH closure occurred in 88% of eyes using the traditional surgical technique and 98% of eyes using the ILM flap-inversion technique, which also resulted in better functional results [8]. Although additional studies have reported better anatomic results with the ILM-inverted technique, recent reports have shown that the outer retina has abnormalities visualized by OCT, which suggests poor functional results [9].

The object of the current study was to compare the anatomic and functional results of idiopathic MH surgery using the standard ILM peeling technique versus the ILM inverted technique or the free-flap technique using OCT angiography (OCTA) images analyzed by ImageJ software (National Institutes of Health, Bethesda, MD).

## Methods

### Study inclusion

This is a retrospective, nonrandomized, observational case series of consecutive patients with idiopathic full-thickness MH treated at the Brazilian Institute of Fight Against Blindness, Assis, Brazil, between May 2017 and November 2018. The Ethics Committee of the Federal University of São Paulo approved the study, which was conducted according to the principles of the Declaration of Helsinki. All patients provided written informed consent for participation in the study.

The inclusion criteria required that all participants undergo a comprehensive ophthalmologic examination, including measurement of the axial length and BCVA using the Early Treatment Diabetic Retinopathy Study chart, dilated indirect slit-lamp biomicroscopy, and SS-OCT. These procedures were performed preoperatively and at 1 week and 1 and 6 months postoperatively. Patients were divided into two groups according to the surgical technique (peeling vs. inverted/free-flap) and followed for at least 6 months.

Patients were excluded if they had a traumatic MH or a history of eye trauma and a diagnosis of glaucoma or any other chronic ocular disease.

Ten participants (10 eyes) were included in the study. Five patients underwent ILM peeling and five underwent the inverted/free-flap technique. Among the latter, three eyes had the inverted technique, whereas two had the free flap. Epidemiologic information such as age, gender, the onset of MH symptoms, and the mean diameter of the initial MH was assessed. OCTA 3.0 x 3.0-mm scans were obtained postoperatively.

### Surgical technique

One experienced retina surgeon (M.M.) performed all the MH surgeries. All phakic patients underwent phacoemulsification with intraocular lens implantation in the capsular bag for better assessment of the peripheral vitreous. A 27-gauge vitrectomy was performed using the DORC System (Dutch Ophthalmic Research Center, Zuidland, The Netherlands) following the conventional sequence: core, posterior vitreous detachment, and peripheral vitreous shaving. BB was used to stain the ILM, and an end-grasping ILM forceps was used for ILM peeling.

The MHs were divided into two treatment groups: the conventional ILM peeling technique and either the inverted ILM flap technique or ILM free-flap transplantation. In the inverted ILM flap technique, the ILM was peeled in the usual manner; however, the ILM remained attached at the edge of the MH and then the peeled ILM was inverted and placed inside the hole. If no residual ILM was observed at the edges of the MH, the ILM transplantation (free-flap) was performed under perfluorocarbon to facilitate the surgical maneuver of ILM positioning over the MH. Finally, careful fluid-air exchange was performed and gas tamponade with perfluoropropane 15%. Patients were advised to maintain prone positioning for 3 days postoperatively.

### Image acquisition

Postoperative OCTA images were obtained using the DRI OCT Triton Swept Source (SS-OCTA) technology (Topcon Corporation, Tokyo, Japan) at least 3 months postoperatively.

SS-OCT has an automated default segmentation technique that shows four different depths of the retinal plexus: the superficial retinal plexus (ILM-inner plexiform layer), the deep retinal plexus (ILM-outer plexiform layer [OPL]), the avascular outer retina (OPL-Bruch’s membrane), and the choriocapillaris [10, 11]. The 3.0 x 3.0-mm OCTA scans were obtained after the instillation of topical anesthetic (proxymetacaine 0.5%), and the patient was asked to look at the fixation point during the image acquisition with the eye tracker on. The quality of the examination was calculated by the SS-OCT itself, according to the centration, fixation, and signal strength.

### Software analysis

**Fig. 1:**
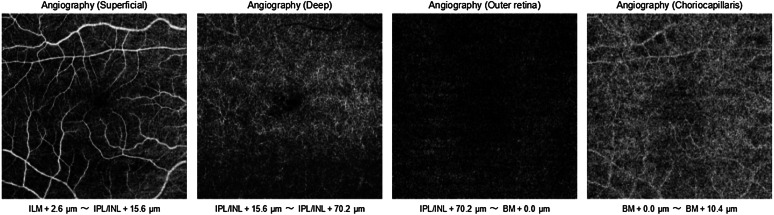
OCTA images of a 62-year-old woman3 months postoperatively with an image quality of 66

**Fig. 2:**
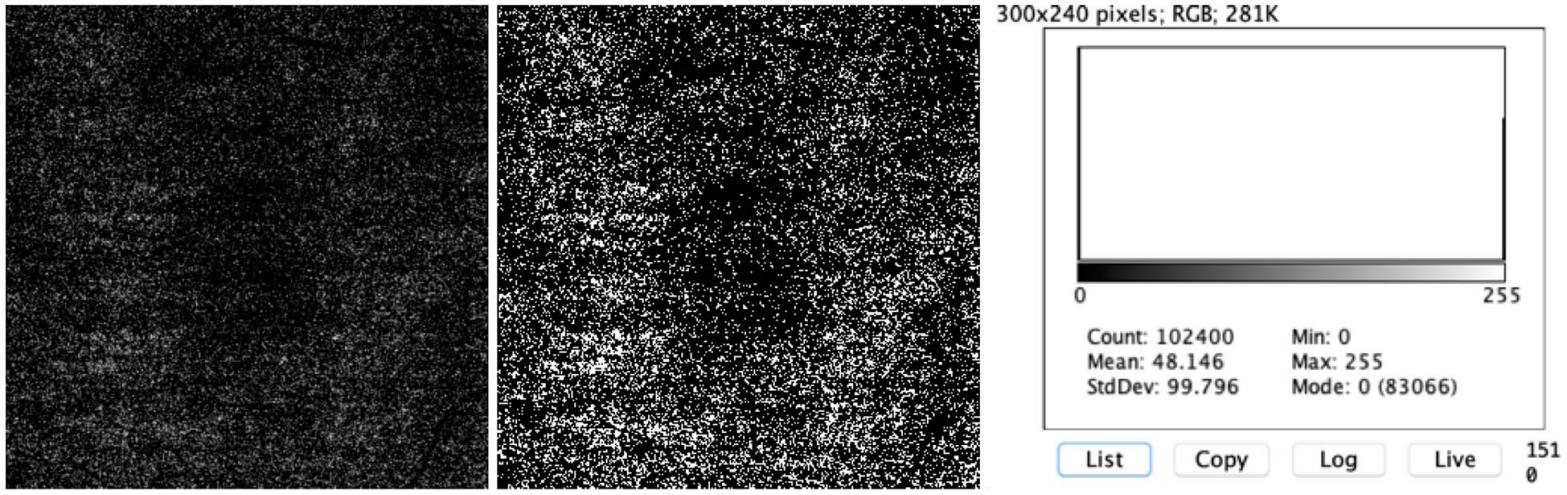
**a** 3.0 x 3.0-mm scan of the outer retina of the same patient (left) and after Otsu binarization (right). The histogram obtained after binarization shows a total count of 102,400 pixels (83,066 black pixels [mode 0] and 19,334 white pixels [mode 255])

**Fig. 3:**
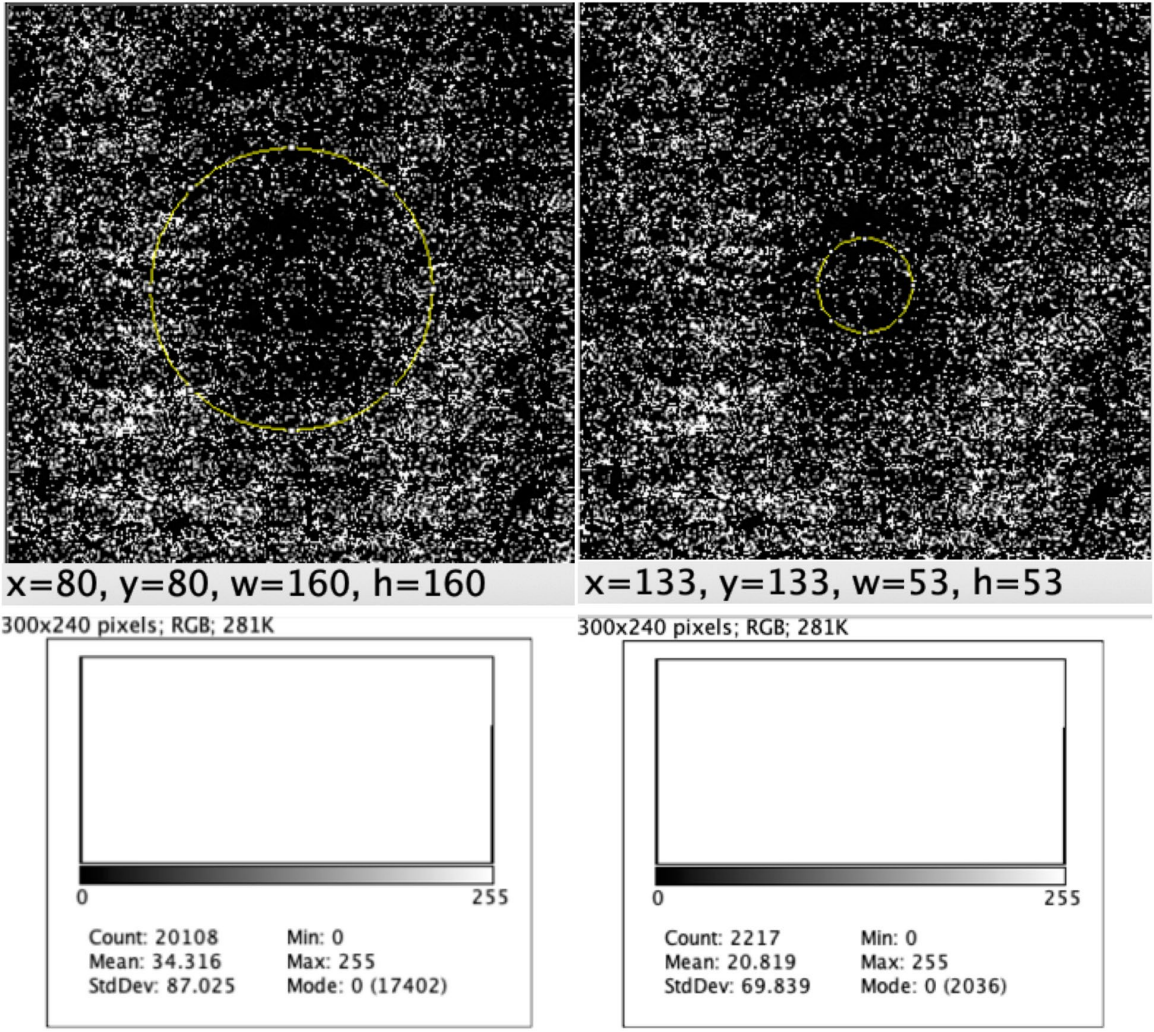
The central 1.5-mm-diameter circle (left) and the 0.5-mm-diameter circle (right). The former has 20,108 pixels (17,402 black pixels and 2706 white pixels). The latter has 2217 pixels (2036 black pixels and 181 white pixels)

The SS-OCTA of the outer retina was analyzed separately (Fig. 1). ImageJ software adjusted the image by an auto-threshold setting, according to Otsu binarization (Fig. 2) [12]. This algorithm can return a single-intensity threshold that separates pixels into two classes, i.e., foreground and background. The software creates a histogram of the pixels and counts the number of black and white pixels after binarization. Three different analyses were performed: within the 3.0 x 3.0-mm area, within a central 1.5-mm-diameter circle, and within a central 0.5-mm-diameter circle (Fig. 3).

The histogram of each selection presented the total count, mean value, standard deviation, number of black pixels (mode 0), and number of white pixels (mode 255).

### Statistical analysis

The mean values between both groups in each situation (3 mm^2^, 1.5-mm-diameter circle, and 0.5-mm-diameter circle) were analyzed by the non-parametric Mann–Whitney test due to the small number of cases in each group.

The linear association between two numerical variables was assessed using the Pearson correlation coefficient.

To analyze the behavior of the BCVA based on the time of evaluation (preoperatively and postoperative), a generalized estimating equation (GEE) model was used with the functions of identity ligation and normal marginal distribution. The GEE approach, which consists of a generalization of generalized linear models, allows the incorporation of the dependency between the observations of the same patient resulting from repeated measures over time. Despite that this model permits normal marginal distribution, the GEE allows relaxation of the supposition of normality in the distribution of dependent variables.

For all statistical tests, P < 0.05 was considered significant. The GEE models were estimated using STATA 12 software (College Station, TX). For other analyzes, the SPSS 20.0 statistical software (SPSS Inc., Chicago, IL) was used.

## Results

Ten eyes of 10 patients (8 women, 2 men; mean age, 66.1 ± 5.8 years (range, 53–73 years) with an idiopathic MH that was closed surgically by the inverted ILM flap technique or ILM peeling were followed by SS-OCT for at least 6 months and enrolled.

The mean time to onset of MH symptoms was 6.4 ± 8.1 weeks in the ILM peeling group and 20.0 ± 5.7 weeks in the inverted flap group (P = 0.026). The mean sizes of the MHs were 423.8 ± 144.2 microns and 889.6 ± 193.8 microns in the ILM peeling and inverted/free-flap groups, respectively; the difference between the two reached significance (P = 0.009). The preoperative BCVAs were 0.7 ± 0.1 logMAR in the peeling group and 1.0 ± 0.l logMAR in the inverted flap group, a difference that did not reach significance (P = 0.055) (Table 1).

**Table 1:** Summary measures of patient characteristics

	ILM peeling (N = 5)	Free flap (N = 5)	P value*
Preoperatively			
MH size (microns)	423.8 ± 144.2	889.6 ± 193.8	0.009
Disease duration (weeks)	6.4 ± 8.1	20.0 ± 5.7	0.026
LogMAR VA	0.7 ± 0.1	1.0 ± 0.3	0.055
Postoperatively			
LogMAR VA	0.3 ± 0.2	0.9 ± 0.4	0.012
%white dots			
3 x 3-mm square	19.9 ± 0.8	20.4 ± 1.7	0.347
1.5-mm-diameter circle	17.1 ± 2.2	14.0 ± 3.0	0.251
0.5-mm-diameter circle	12.3 ± 4.8	5.6 ± 2.1	0.028

The data are expressed as the mean ± SD

*Mann–Whitney test

Thus, the inverted/free flap group had higher mean values than the peeling group for the time of symptom onset, MHs, and the trend of worsening BCVA, indicating worse functional prognosis for these patients. Corroborating the BCVA result, the inverted/free flap group also had a lower percentage of white dots at the pixelized binarization.

**Fig. 4:**
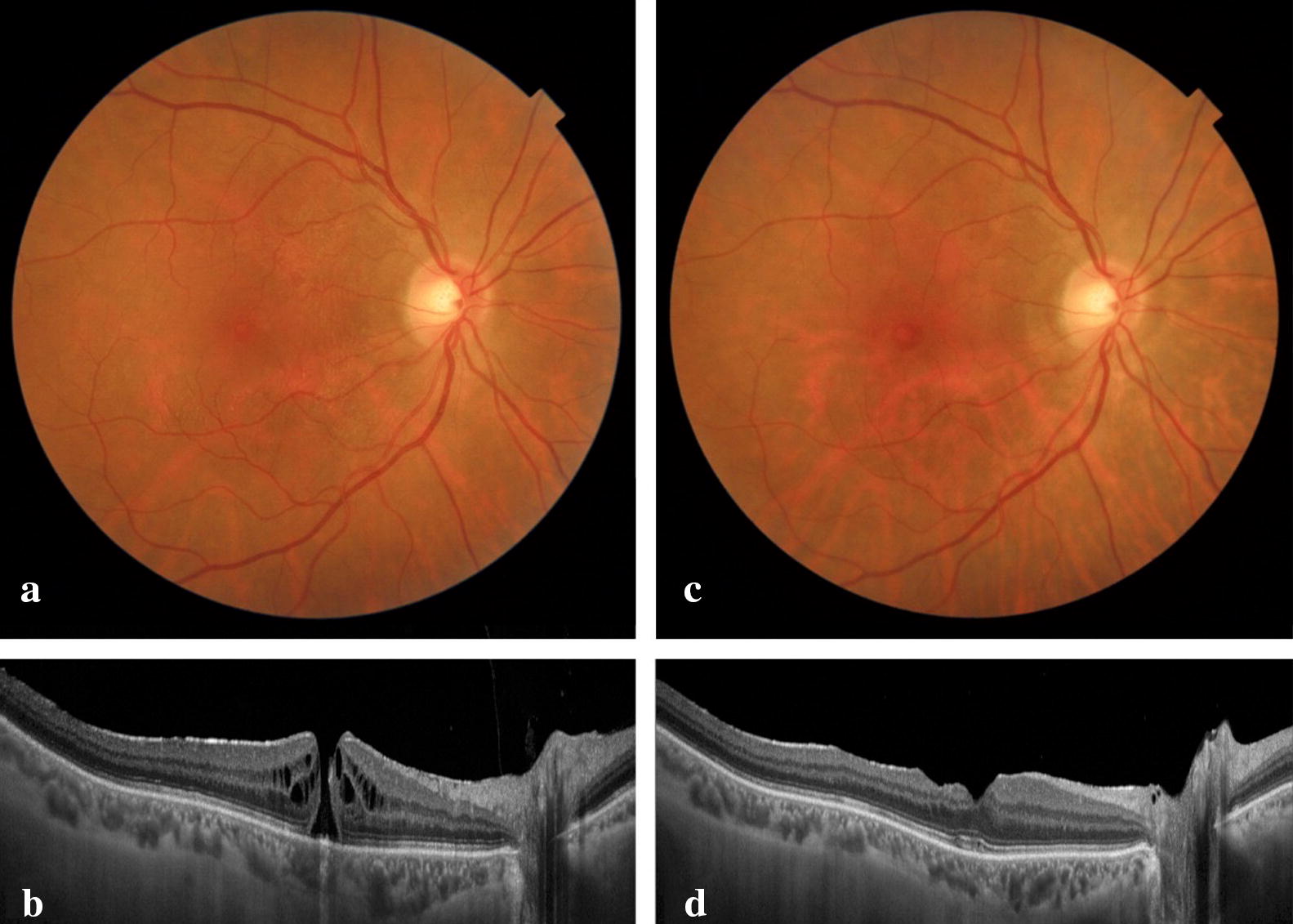
**a** A fundus photograph of a 65-year-old woman with a small MH in the right eye. **b** The minimal MH diameter is 124 microns. The preoperative logMAR BCVA is 0.6 and she had symptoms for 8 weeks. **c** A fundus photograph obtained 6 months postoperatively in which the ILM peeling technique was performed. The MH is closed. **d** Spectral-domain OCT shows the inner segment/outer segment junction in the fovea with a restored external limiting membrane. The visual acuity is 0.1

**Fig. 5:**
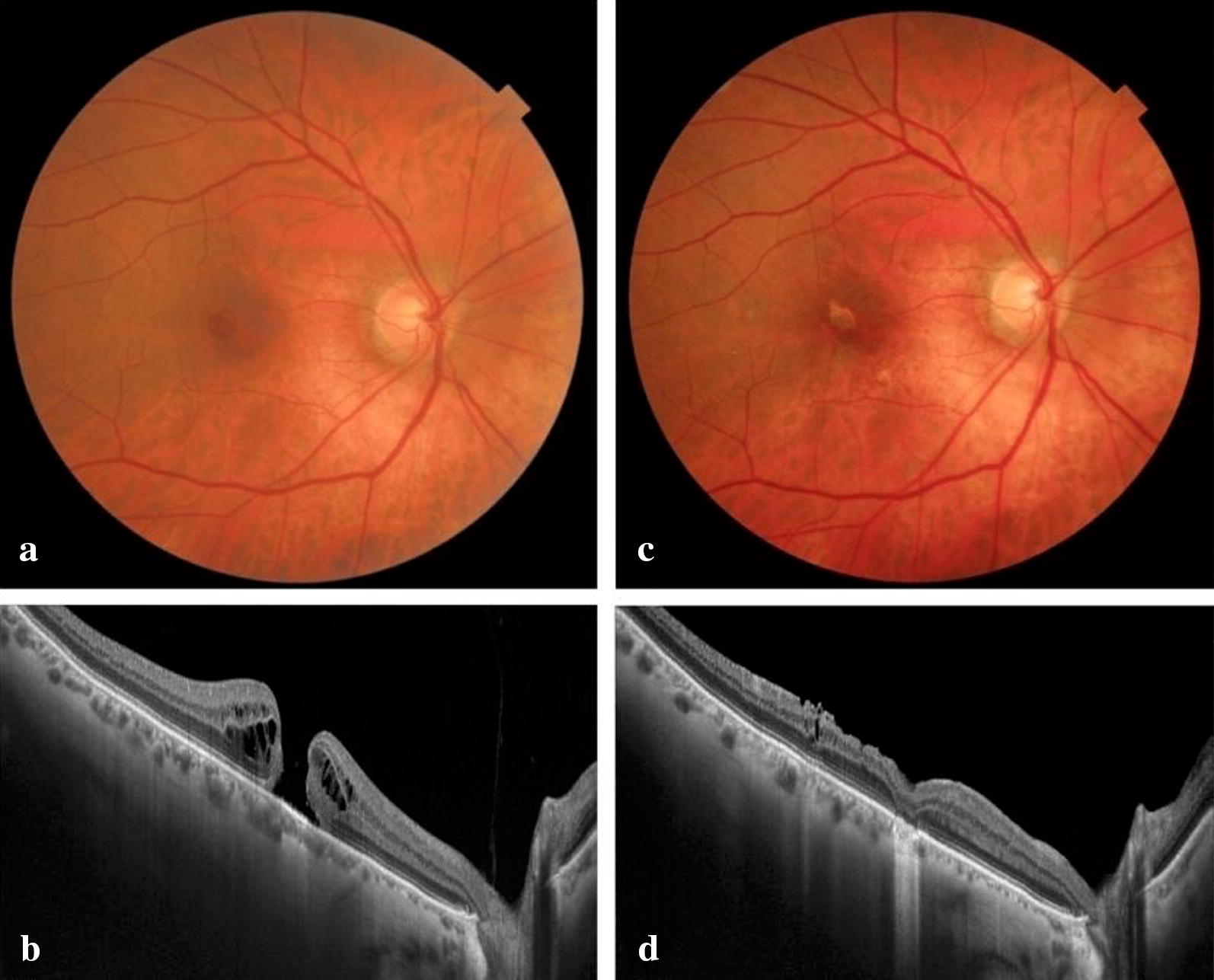
**a** A fundus photograph of a 73-year-old woman with a large MH in the right eye. **b** The minimal MH diameter is 463 microns. The preoperative logMAR BCVA is 1.6; the patient had symptoms for 16 weeks. **c** A fundus photograph obtained 6 months postoperatively in which the free-flap technique was performed. The MH is closed. **d** Spectral-domain OCT shows foveal loss of the outer segments (external limiting membrane, inner segment/outer segment junction, and RPE) and hence the presence of reverse shadowing. The final visual acuity is 1.0

All the MHs closed in the ILM peeling group (Fig. 4) and inverted/free-flap group (Fig. 5).

The postoperative 3.0 x 3.0-mm OCTA images from all 10 eyes were analyzed. This area is related to the original software of the device and represents the foveal, macular, and perimacular regions. In the 3.0-mm^2^ area, the percentages of white pixels ranged from 18.88% to 20.91% in the peeling group and from 17.78% to 22.08% in the inverted flap group. The means ± standard deviations (SDs) of the percentages of the white dots in the peeling group and the flap group were 19.9% ± 0.8% and 20.4% ± 1.7%, respectively (P = 0.347).

To reduce the area of analysis to approximate the previous MH site, a central 1.5-mm diameter circle also was studied. In the peeling group, the percentage of white pixels ranged from 13.46% to 18.86%, and in the inverted flap group, the percentages ranged from 11.32% to 18.89%. The respective mean ± SDs were17.1% ± 2.2% and 14.00% ± 3.0% (P = 0.251).

The last analysis was performed in the foveal area in a central 0.5-mm-diameter circle. In the peeling group, the percentages of white pixels ranged from 7.22% to 18.40% and in the inverted flap group from 3.65% to 8.93% (Table 2).

**Table 2:** ImageJ analysis of the 0.5-mm-diameter center circle

Eye	Technique	Total count	Black (#0)	% Black	White (#255)	% White	Mean	Standard deviation
1	Peeling	2217	2057	92.78%	160	7.22%	18.403	66.001
2	Peeling	2217	1809	81.60%	408	18.40%	46.928	98.838
3	Peeling	2217	1868	84.26%	349	15.74%	40.142	92.891
4	Peeling	2217	1957	88.27%	260	11.73%	29.905	82.064
5	Peeling	2217	2036	91.84%	181	8.16%	20.819	69.839
6	Flap	2217	2072	93.46%	145	6.54%	16.678	63.06
7	Flap	2217	2119	95.58%	98	4.42%	11.272	52.427
8	Flap	2217	2019	91.07%	198	8.93%	22.774	72.74
9	Flap	2217	2136	96.35%	81	3.65%	9.317	47.854
10	Flap	2217	2117	95.49%	100	4.51%	11.502	52.934

The 0.5-millimeter area is related to the fovea area and, therefore, provides the most reliable evaluation of the RPE/photoreceptors. The mean ± SDs determined using the ImageJ software in the peeling group were 12.3% ± 4.8% versus 5.6 ± 2.1% in the flap group (P = 0.028).

**Fig. 6:**
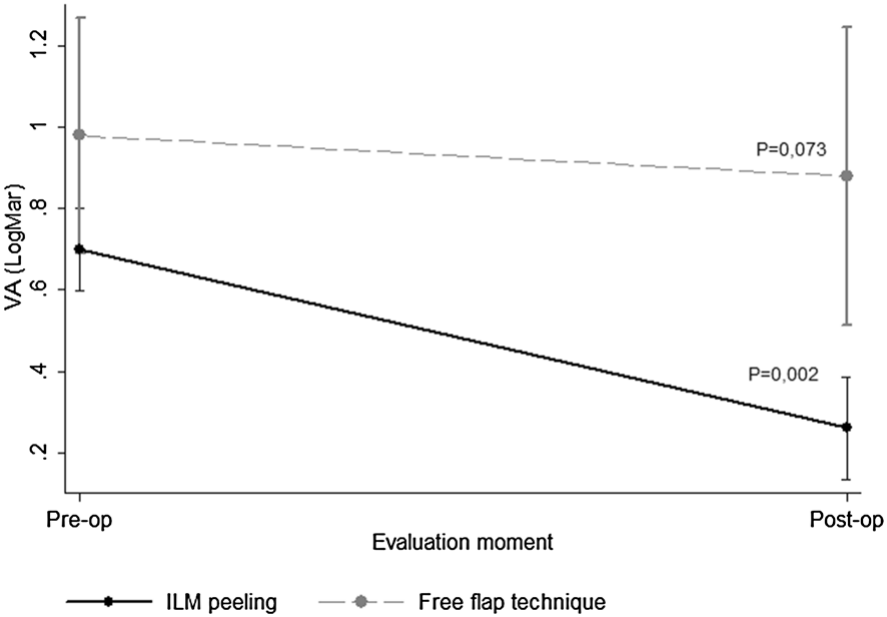
The GEE analysis shows stable VA in the free-flap group with the worst VA, and the peeling group had significant (P = 0.002) improvement of the BCVA

The preoperative BCVAs did not differ significantly (P = 0.055) between the groups based on the GEE analysis; however, the difference between the postoperative BCVAs reached significance (P = 0.002). Comparison of the postoperative and preoperative BCVAs in the peeling and flap groups showed that the BCVAs in the peeling group were higher, although the difference did not reach significance (P = 0.162). The initial BCVAs in the peeling group ranged from 0.6 to 0.9 logMAR (mean, 0.7 ± 0.1) and the postoperative BCVA improved from 0.1 to 0.5 logMAR (mean, 0.3 ± 0.2). Besides, the initial BCVAs in the inverted-flap group ranged from 0.7 to 1.6 logMAR (mean,1.0 ± 0.3) and the final BCVA improved from 0.5 to 1.6 (mean,0.9 ± 0.4) (Table 3, Fig. 6).

**Table 3:** GEE model results

	Peeling (N = 5)	Free flap (N = 5)	P value*
Preoperative VA	0.7 ± 0.1	1.0 ± 0.3	0.073
Postoperative VA	0.3 ± 0.2	0.9 ± 0.4	0.002
Postoperative–preoperative VA	−0.4 ± 0.2	−0.1 ± 0.5	0.162

The data are expressed as the mean ± SD

*Descriptive level of the GEE model

**Fig. 7:**
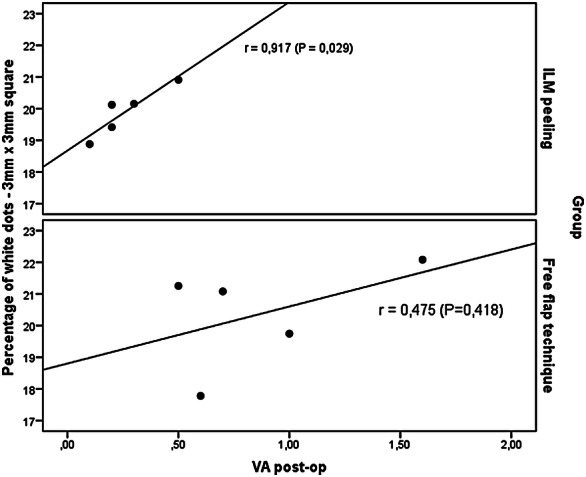
Scatter plot between the postoperative VA and the percentage of white dots in a 3 x 3-mm square

A significant correlation was observed between the postoperative VA and the percentage of white dots in the 3x3-mm square in the peeling group (r = 0.917, P = 0.029) (Table 4, Fig. 7), indicating that the better the postoperative VA, the higher the percentage of white dots in the ImageJ analysis. In the inverted/free-flap group, no significant correlation was observed. For the other circular areas, no significant correlations were observed in either group.

**Table 4:** Pearson correlation coefficient between the VA (logMAR) and percentage of white dots

White dot percentage	Pearson’s correlation	
	Estimate	P value
ILM peeling (N = 5)		
3 x 3-mm square	0.917	0.029
1.5-mm-diameter circle	0.778	0.121
0.5-mm-diameter circle	0.206	0.739
Free flap (N = 5)		
3x 3-mm square	0.475	0.418
1.5-mm-diameter circle	0.227	0.714
0.5-mm-diameter circle	0.014	0.983

## Discussion

OCTA has been used previously to study MHs preoperatively and postoperatively. Initially, some authors have reported that the foveal avascular zone returned to near-normal size after successful hole closure [13, 14]. The macular region was also reported to be displaced nasally, probably due to contraction after ILM removal [15, 16].

Some authors reported that the healing of the inner segment/outer segment junction varied among MHs that had closed and hypothesized that the integrity of that junction was related to a visual electrical signal in the outer retina [17]. Previous histopathologic findings showed glial cell proliferation pushed healthy photoreceptors to the central fovea [18].

The study of OCTA images of full-thickness MHs revealed decreased vessel density around the MH in the deep retina, which may be correlated with unsuccessful postoperative results. When parafoveal cystoid spaces are present, there is some perfusion around the MH, even if the vessels are pushed aside, and the reperfusion of these vessels may lead to better surgical results [19]. Additional large prospective studies are necessary to validate these hypotheses.

A prospective case series study compared the morphologic and vascular OCTA features before and after conventional peeling of ILMs in eyes with idiopathic MHs. The postoperative findings were increased foveal vascular density in the superficial plexus, increased flow area in the choriocapillaris, and a reduced foveal avascular zone. These anatomic and hemodynamic changes implied that the recovery process of the inner retina has neuronal and vascular plasticity [20].

In this case series, the initial BCVA in the peeling group had low variability (0.7 ± 0.1) and the final BCVA was significantly (P = 0.002) better (0.3 ± 0.2) than in the inverted-flap group (0.9 ± 0.4). Because the time of symptom onset (P = 0.026), and mean MH size (P = 0.09) were significantly higher in the inverted/free-flap group, it was impossible to compare one technique with the other directly. It is reasonable that the worse results in the inverted flap or the free-flap technique also were related to the natural history of chronic and larger holes and not the surgical technique itself; however, both factors may be involved.

**Fig. 8:**
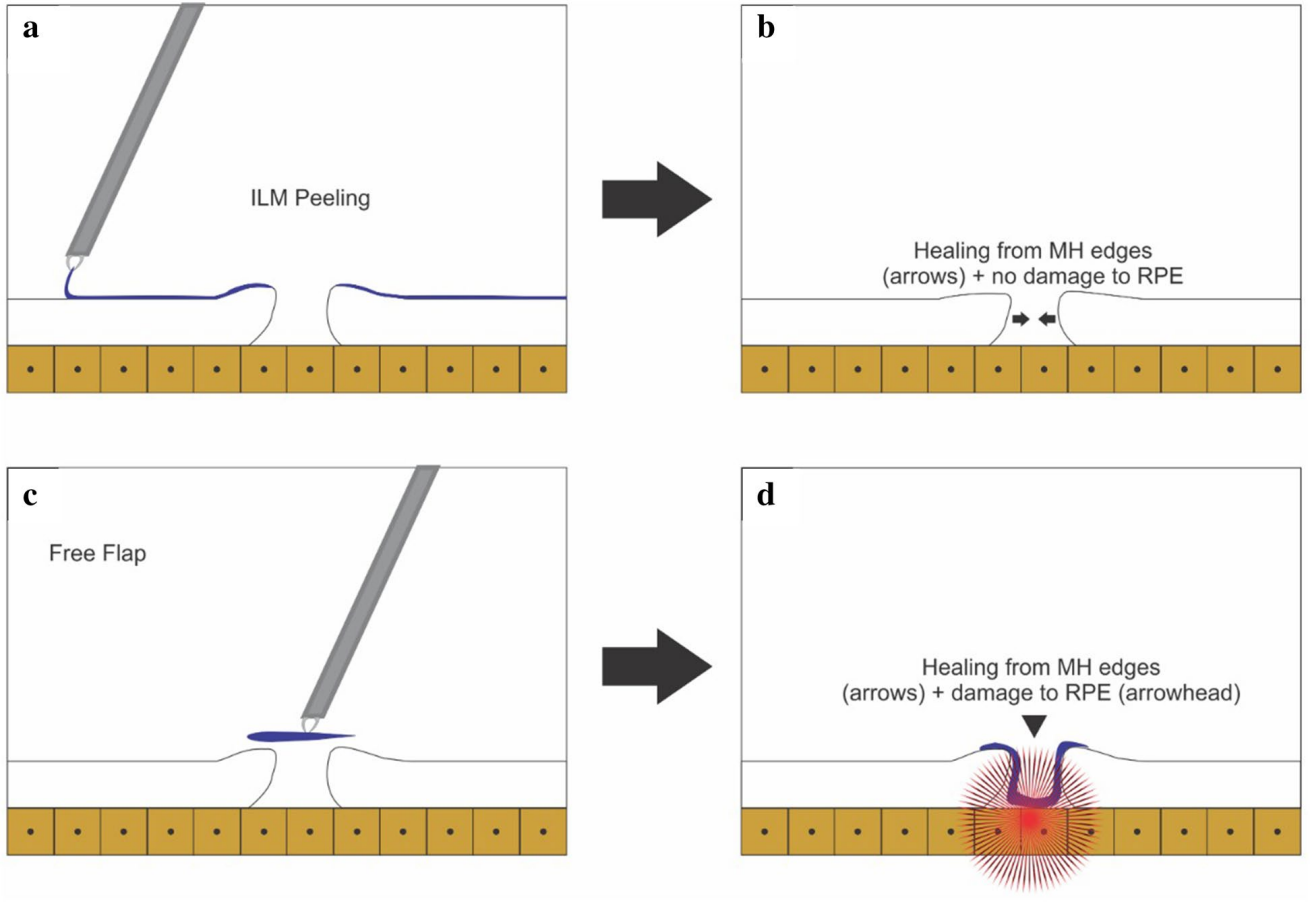
**a, b** Schematic drawing of ILM peeling surgery and the healing process through the MH edges without damaging the RPE. **c, d** Schematic drawing of the free-flap surgery and healing process with possible damage to the RPE by compression caused by the ILM flap

We hypothesized that the contact between the ILM and the RPE aided by the compression of the ILM (inverted or free-flap) against the RPE may cause RPE damage and poor final BCVA in inverted/free-flap techniques (Fig. 8b) compared with the ILM technique (Fig. 8a); however, it is impossible to reach a definitive conclusion based on the design of this current study and the baseline differences in both groups.

The methodology of the current study was based on the correlation of the final BCVA with the OCTA findings using the ImageJ software analysis of the conventional ILM peeling technique and the inverted/free-flap technique in eyes with an idiopathic MH.

The worse final BCVA in the inverted/free-flap technique with fewer associated white dots in the ImageJ analysis of the outer retinal layer showed a relation between the poor postoperative BCVA that probably was related to loss of RPE/photoreceptors cells and/or the poor natural history of this group.

To the best of our knowledge, this is the first study in the medical literature that shows the applicability of the grayscale images of ImageJ software from the OCTA analysis data of the outer retina after MH surgery and a possible correlation with the BCVA postoperatively (Medline search, November 18, 2019). This is an innovative, non-invasive, and simple methodology for evaluating the anatomy of the outer retina that may provide functional implications in MH surgeries.

Despite the small number of eyes, retrospective study design, and no similarities between the preoperative MH characteristics, we hypothesized that the surgical techniques of MH surgery using the inverted flap and/or free flap may result in higher anatomic success rates despite the lower functional results; for this reason, such surgical techniques should be reserved for chronic or refractory MHs (Fig. 8b).

## Conclusions

In conclusion, the current study reported on a simple methodology of imaging analysis to separate white and black pixels using the non-invasive and simple OCTA technology and hypothesized that more white pixels corresponded to viable cellularity after MH surgery. Prospective studies with more cases that analyze the distinct surgical techniques performed to treat comparable MHs using this innovative technology are necessary to validate this hypothesis.

## Data Availability

The datasets used and/or analysed during the current study are available from the corresponding author on reasonable request.

## Abbreviations

MH: Macular hole
SS-OCT: Swept-source optical coherence tomography
OCTA: Optical coherence tomography angiography
BCVA: Best-corrected visual acuity
PPV: Pars plana vitrectomy
ILM: Internal limiting membrane
BB: Brilliant blue
GEE: Generalized estimating equation
RPE: Retinal pigment epithelium
SD: Standard deviation
logMAR: Logarithm of the minimum angle of resolution
OPL: Outer plexiform layer
